# Supplementary material for: Femtosecond formation dynamics of the spin Seebeck effect revealed by terahertz spectroscopy
Source: Nat Commun. 2018 Jul 24;9:2899. doi: 10.1038/s41467-018-05135-2 (PMC6057952; doi:10.1038/s41467-018-05135-2)
Supplement: Supplementary file 1 — Supplementary Information [file 41467_2018_5135_MOESM1_ESM.pdf]

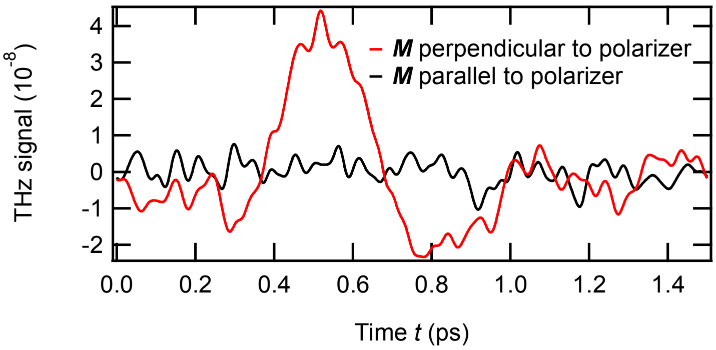

Supplementary Figure 1 | Polarization state of the emitted THz radiation. THz signal components with polarization perpendicular and parallel to the in-plane sample magnetization. For the polarization analysis, a wire-grid polarizer was placed behind the YIG(3 μm)|Pt(5.5 nm) sample, and the in-plane sample magnetization was set parallel and perpendicular to the polarizer direction. Data were low-pass-filtered with a Gaussian filter of 15 THz FWHM centered at 0 THz.

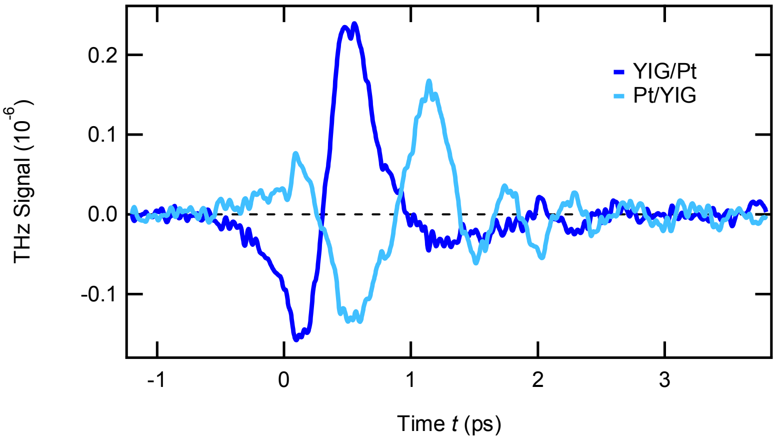

Supplementary Figure 2 | Effect of reversing the YIG|Pt bilayer. Upon reversing a GGG(substrate)|YIG(3 μm)|Pt(5 nm) sample, we observed reversal of the signal $S_{-}(t)$ odd in the sample magnetization. For the reversed sample, the propagation of the THz pulse through the GGG substrate led to significant distortion of the THz signal. To correct for the different dispersion of the GGG substrate for THz and optical radiation, waveforms from YIG/Pt were shifted to earlier time delays by 1 ps.

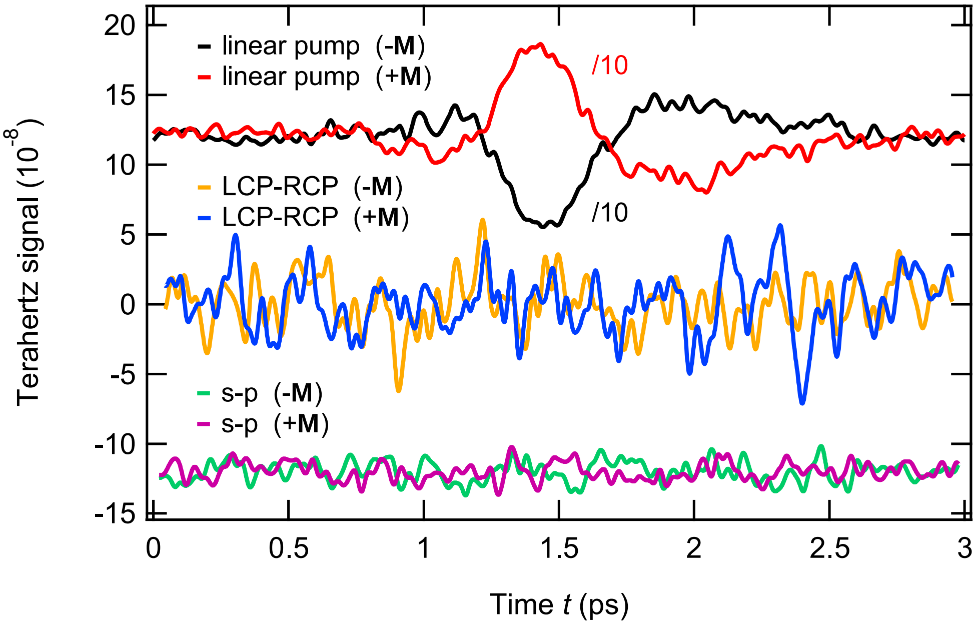

Supplementary Figure 3 | Impact of pump polarization. Upper curves: p-polarized pump. Middle: difference signal of left-handed circularly polarized (LCP) minus right-handed circularly polarized (RCP) pump polarization. Lower: difference signal of s- minus p-polarized pump pulses. The sample was YIG(3 µm)|Pt(5.5 nm).

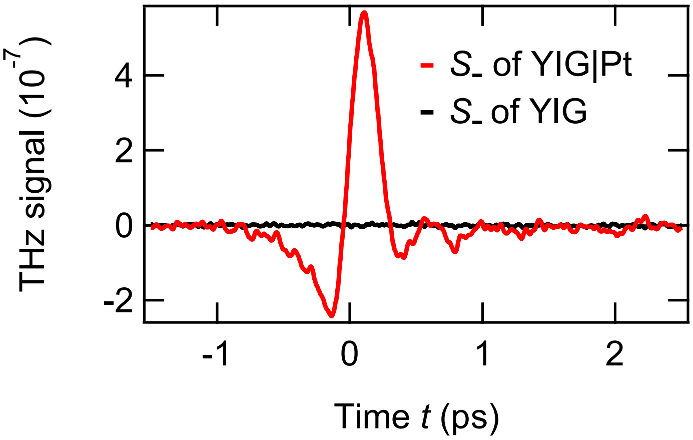

Supplementary Figure 4 | THz emission from pure YIG. THz signal component $S_-(t)$ from YIG(3 µm)|Pt(5.5 nm) (red curve) and YIG(10 µm) without metal coating on diamond substrate (black curve).

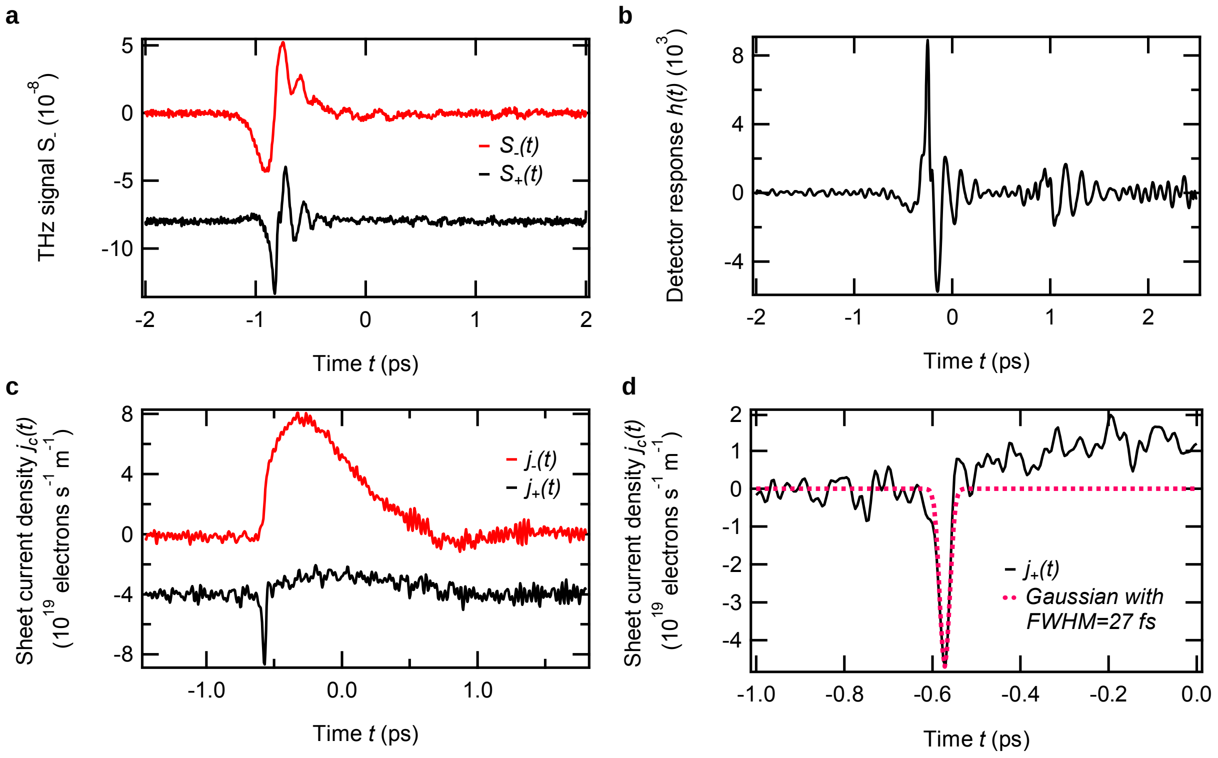

Supplementary Figure 5 | Current extraction and time resolution. (**a**) THz emission signals $S_{\pm}(t)$ even and odd in the in-plane YIG magnetization M from a YIG(3 µm)|Pt(5.5 nm) sample. (**b**) Measured detector response function using a reference emitter [GaP(110) crystal of 50 µm thickness] under similar experimental conditions. (**c**) Extracted sheet current densities $j_{\pm}(t)$ even and odd in the sample magnetization. (**d**) Fit of a Gaussian to the temporally sharpest feature of $j_{+}(t)$, yielding a full width at half maximum of 27 fs.

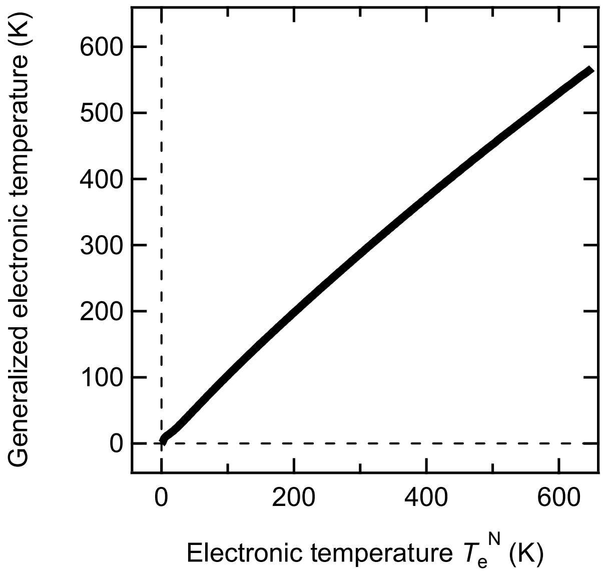

Supplementary Figure 6 | Generalized temperature for Pt. Generalized electronic temperature $\tilde{T}_e^N$ of Pt as calculated from Eq. (3) (main text) for a Fermi-Dirac distribution of varying temperature T_e^N.

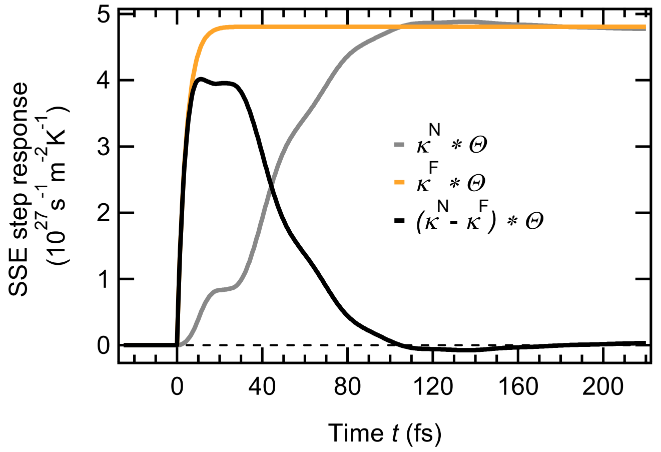

Supplementary Figure 7 | SSE step response. SSE currents $\int_0^t \mathrm{d}t' \, \kappa^{\mathrm{N}}(t')$ and $\int_0^t \mathrm{d}t' \, \kappa^{\mathrm{F}}(t')$ (grey and yellow curve, respectively) induced by the same temporally step-like temperature increase $\Theta(t)$ at $t = 0$ in both layers. Their difference, which equals the total response, is also shown (black curve). To obtain an absolute ordinate scaling in agreement with the experiment, an interfacial exchange constant of $J_{\mathrm{sd}} = 2 \text{ meV}$ was used.

Parameter	Value	Reference
Density of states at Fermi energy of Pt	2 states atom^{-1} eV^{-1}	[1]
Density of states at Fermi energy of Cu	0.3 states atom^{-1} eV^{-1}	[2]
Fermi velocity of Pt	$0.5\cdot10^{6}$ m s^{-1}	[3]
Fermi velocity of Cu	$1.6\cdot10^{6}$ m s^{-1}	[4]
Cubic lattice constant of YIG	1.24 nm	[5]
YIG magnetization per unit cell (300 K)	$7.2\hbar$	Result of atomistic spin-dynamics simulations by J. Barker
Pump-pulse duration	10 fs	Measured
Absorbed pump-pulse fluence	1.185 J m^{-2}	Measured
Conductivity of Pt	$(4+0.1i)\cdot10^{6}$ S m^{-1}	Measured
Infrared refractive index of YIG	5	[6]
Infrared refractive index of GGG	3.5	[7]

Supplementary Table 1 | Material parameters relevant for numerical estimates.

Parameter	Value	Reference
Speed of sound	3260 m s^{-1}	[8]
Debye temperature	240 K	[1]
Density of states		[1]
Lattice constant	3.92 Å	[1]

Supplementary Table 2 | Material parameters of Pt relevant for the simulations.

Supplementary references

[1] Lin, Z., Zhigilei, L.V. & Celli, V. Electron-phonon coupling and electron heat capacity of metals under conditions of strong electron-phonon nonequilibrium. *Phys. Rev. B* **77**, 075133 (2008).

[2] Moruzzi, V.L., Janak, J.F. & Williams, A.R. *Calculated electronic properties of metals*. Elsevier, 2013.

[3] Ketterson, J. B. et al. Fermi velocity and Fermi radius in platinum. *Solid State Commun.* **6**, 851-854 (1968).

[4] Ashcroft, N.W.& Mermin, N.D. *Solid state physics*. Holt, Rinehart and Winston, 1976.

[5] Douglass, R.L. Spin-wave spectrum of yttrium iron garnet. *Phys. Rev.* **120**, 1612 (1960).

[6] Hofmeister, A.M. & Campbell, K.R. Infrared spectroscopy of yttrium aluminum, yttrium gallium, and yttrium iron garnets. *J. Appl. Phys.* **72**, 638-646 (1992).

[7] Lal, K. & Jhans, H.K. The dielectric constant of gadolinium gallium garnet and α-Al_2O_3 single crystals. *J. Phys. C: Solid State Phys.* **10**, 1315 (1977).

[8] Lide, D.R., *CRC Handbook of Chemistry and Physics*. CRC Press, 2004.
